# Infection-Induced Interaction between the Mosquito Circulatory and Immune Systems

**DOI:** 10.1371/journal.ppat.1003058

**Published:** 2012-11-29

**Authors:** Jonas G. King, Julián F. Hillyer

**Affiliations:** Department of Biological Sciences, Vanderbilt University, Nashville, Tennessee, United States of America; Stanford University, United States of America

## Abstract

Insects counter infection with innate immune responses that rely on cells called hemocytes. Hemocytes exist in association with the insect's open circulatory system and this mode of existence has likely influenced the organization and control of anti-pathogen immune responses. Previous studies reported that pathogens in the mosquito body cavity (hemocoel) accumulate on the surface of the heart. Using novel cell staining, microdissection and intravital imaging techniques, we investigated the mechanism of pathogen accumulation in the pericardium of the malaria mosquito, *Anopheles gambiae*, and discovered a novel insect immune tissue, herein named periostial hemocytes, that sequesters pathogens as they flow with the hemolymph. Specifically, we show that there are two types of endocytic cells that flank the heart: periostial hemocytes and pericardial cells. Resident periostial hemocytes engage in the rapid phagocytosis of pathogens, and during the course of a bacterial or *Plasmodium* infection, circulating hemocytes migrate to the periostial regions where they bind the cardiac musculature and each other, and continue the phagocytosis of invaders. Periostial hemocyte aggregation occurs in a time- and infection dose-dependent manner, and once this immune process is triggered, the number of periostial hemocytes remains elevated for the lifetime of the mosquito. Finally, the soluble immune elicitors peptidoglycan and β-1,3-glucan also induce periostial hemocyte aggregation, indicating that this is a generalized and basal immune response that is induced by diverse immune stimuli. These data describe a novel insect cellular immune response that fundamentally relies on the physiological interaction between the insect circulatory and immune systems.

## Author Summary

Mosquitoes transmit diseases such as malaria, dengue fever, West Nile virus and lymphatic filariasis. A mosquito initially acquires a pathogen when she ingests a blood meal from an infected person or animal. Then, after a period of development and/or replication in the mosquito gut, the pathogen enters the hemocoel (body cavity) and undergoes an obligate migration to the salivary glands (the destination for viruses and protozoans) or the mouthparts (the destination for larger worms). During this migration, pathogens are subject to two potentially antagonistic mosquito forces: immune responses and circulatory currents. In this study, we examined the physiological interactions between the mosquito immune and circulatory systems. We show that when mosquitoes are infected with bacteria or malaria parasites, mosquito immune cells (hemocytes) migrate to the areas surrounding the valves of the heart. At these areas of rapid and dynamic hemolymph (mosquito blood) flow, hemocytes swiftly phagocytose and kill pathogens. These experiments describe a novel and basal insect immune response that fundamentally relies on the physiological interaction between the mosquito circulatory and immune system. Furthermore, because traversal of the hemocoel is required for pathogen transmission, this new knowledge could be used in the development of novel disease control strategies.

## Introduction

**Figure 1:**
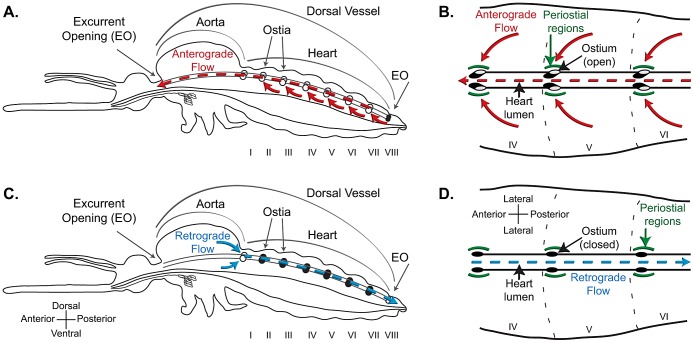
Structural mechanics of the mosquito dorsal vessel. A–B. Lateral view of an entire mosquito (A) and dorsal view of three abdominal segments (B; segments IV, V and VI), illustrating that the dorsal vessel is a tube-like structure that is divided into a thoracic aorta and an abdominal heart. During anterograde heart flow (red arrows), hemolymph enters the heart through paired ostia (valves) located in the anterior portion of each abdominal segment, and is propelled to the head, where it exits the dorsal vessel through an excurrent opening. The areas surrounding the ostia are referred in this study as the periostial regions (green arcs). C–D. During retrograde heart flow (blue arrows), hemolymph enters the heart through a single pair of ostia located at the thoraco-abdominal junction, and is propelled to the posterior abdomen, where it exits the dorsal vessel through a paired excurrent opening. For a detailed description of the mosquito heart, see Glenn et al. [3].

Pathogens transmitted by mosquitoes must traverse the insect's open body cavity (hemocoel) during their journey from the midgut to the salivary glands, and this obligate migration places them in direct contact with the insect's circulatory and immune systems. The insect circulatory system consists of hemolymph (blood), the hemocoel, and pulsatile organs, of which the dorsal vessel is the most important [1]. The dorsal vessel extends along the dorsal midline of the insect and is anatomically divided into a thoracic aorta and an abdominal heart. In adult mosquitoes, the heart drives hemolymph propulsion by sequentially contracting in a wave-like manner, with the contractile waves periodically alternating between propagating in the anterograde (toward the head) and retrograde (toward the posterior abdomen) directions (Figure 1) [2], [3]. When the heart contracts in the anterograde direction, hemolymph enters the lumen of the vessel through six pairs of incurrent ostia (valves) located in the anterior portion of abdominal segments 2 through 7 and exits through an excurrent opening located in the head region [2], [3]. When the heart contracts in the retrograde direction, hemolymph enters the vessel through a single ostial pair located at the thoraco-abdominal junction and exits through an excurrent opening located in the terminal abdominal segment. Variants of this arrangement are seen in all insects and similar systems are present in all arthropods [4], conclusively supporting its ancient origin.

While insect circulatory processes have been primarily studied for their role in transporting nutrients, wastes and signaling molecules, one aspect that has been overlooked is the relationship between hemolymph circulation and immune responses. The insect immune system relies on innate reactions to fight pathogens and involves both cellular and humoral components [5]–[7]. To date, studies on insect immunity have focused primarily on dissecting the molecular bases of immunity and on understanding the cellular biology of hemocytes (immune cells). These experiments have largely assessed immune responses at single points in time, and have used methods that require insect death during the extraction of hemocytes or other tissues. In the hemocoel, hemocytes, humoral immune factors and pathogens exist in contiguous association with the insect's circulatory organs and have likely shared such an existence throughout the course of arthropod evolution. Because this association occurs in a fluid and dynamic space, we hypothesized that hemolymph currents influence the temporal and spatial control of anti-pathogen responses. Furthermore, given that the closed circulatory and lymphatic systems of vertebrate animals are integrally associated with immune surveillance [8], [9], we hypothesized that coordinated interactions between the insect's open circulatory system and immune system are essential for effective insect immune responses. Whether this interaction occurs remains unexplored in any insect, and mosquitoes are an exceptional model for its investigation because: (1) physiological interactions between mosquitoes and a taxonomically diverse array of pathogens have been explored [10]–[13], (2) the mosquito circulatory system has been well characterized [2], [3], (3) the phylogenetic distance between mosquitoes and *Drosophila melanogaster* provides a unique perspective on the evolution of the insect immune and circulatory systems, and (4) it has been proposed that mosquito immune responses could be harnessed for the control of mosquito borne disease [14]–[16].

We have reported that during the course of an infection in the malaria mosquito, *Anopheles gambiae*, pathogens accumulate in discrete foci along the surface of the mosquito heart [11]. Specifically, pathogens accumulate as flanking lines on the anterior portion of each abdominal segment in areas that match the location of the heart's ostia [3]. Here, we used intravital imaging and microdissection techniques to show that during the course of bacterial and malarial infections, mosquito hemocytes migrate to the areas surrounding the heart's ostia, defined here as the periostial regions (Figure 1), where they bind the musculature and each other, and engage in the rapid phagocytosis of pathogens. This invertebrate cellular immune response integrally involves hemolymph circulation and the heart, and relies on the physiological interaction between the mosquito circulatory and immune systems, supporting their co-adaptation to counter pathogens. Furthermore, this discovery suggests that many studies into mosquito immune responses have underestimated the role hemocytes play in controlling infection.

## Results

### Pericardial cells (PCs) flank the mosquito heart but do not phagocytose pathogens

**Figure 2:**
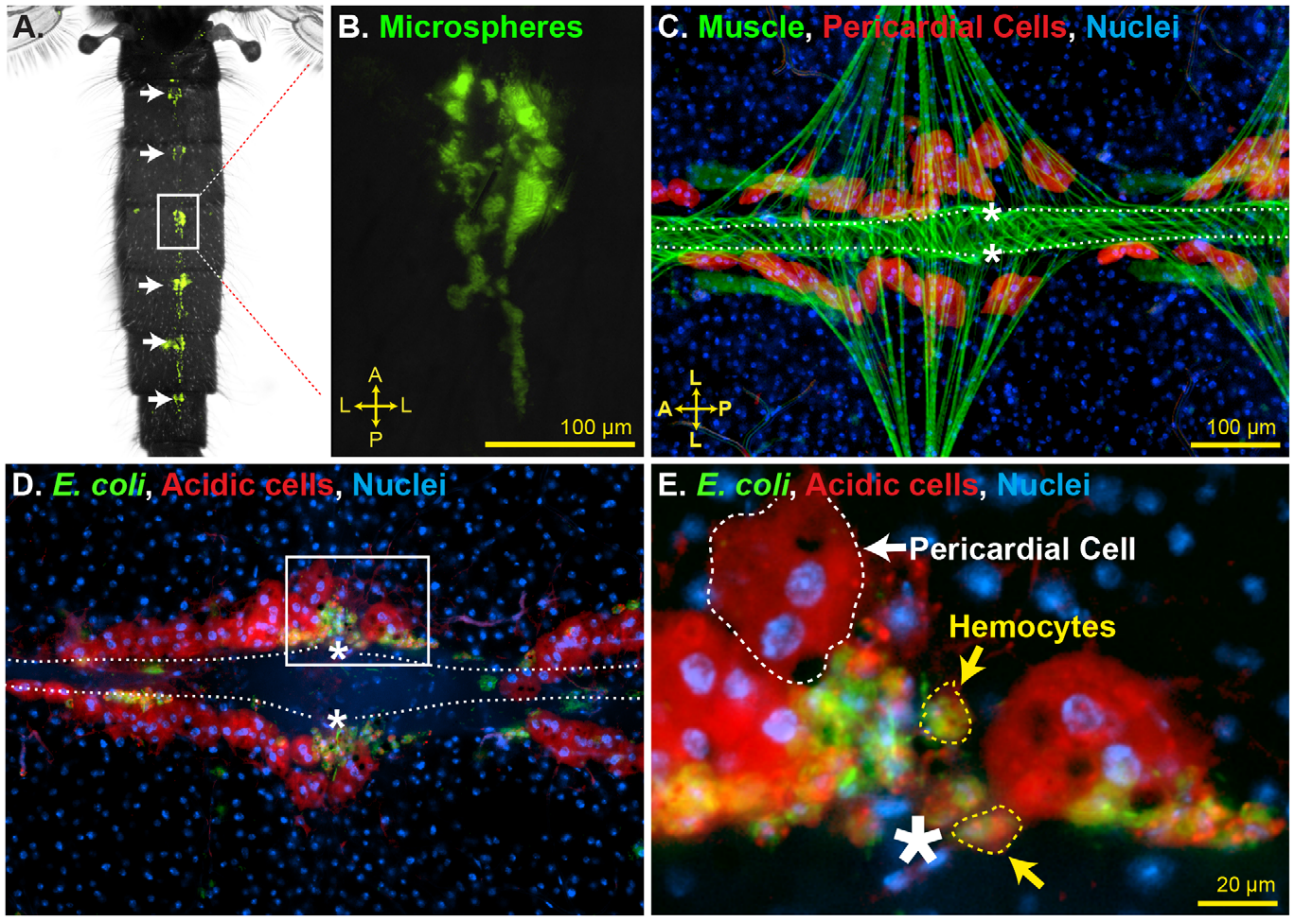
Identification of periostial immune foci. (A–B) Top down view of a mosquito injected with green fluorescent microspheres 24 h earlier. Microspheres accumulate in the periostial regions of each abdominal segment (arrows and rectangle, which is magnified in B). (C) Pericardial cells (568 nm-IgG; red) flank the heart (phalloidin; green tube that extends horizontally), including the periostial regions (asterisks). (D–E) At 24 h post-infection with GFP-*E. coli*, numerous acidic cells (LysoTracker Red; red) are associated with the heart. PCs do not phagocytose pathogens. Instead, smaller acidic cells located at the periostial regions intensely phagocytose bacteria (e.g., rectangle in D, which is magnified in E). A, anterior; P, posterior; L, lateral; asterisks, location of the ostia; dotted lines, outline of the heart. In panels C–E, Hoechst 33342 (blue) was used as a nuclear stain.

The initial goal of this study was to investigate whether pathogen accumulation around the dorsal vessel is due to a physical barrier or due to an active immune response (Figure 2A–B) [11]. Hemolymph in the abdominal cavity flows dorsally toward the ostia [2], [3], and as part of this flow it is possible that pathogens could become stuck in the fenestrated dorsal diaphragm or at the narrow openings of the ostia. Alternately, pathogens could be sequestered as part of an active immune response, and an earlier study suggested that this would most likely be a PC-mediated immune response [11]. To distinguish between these two scenarios we developed two novel methods for the labeling of PCs in vivo. The first employs the injection of AlexaFluor-conjugated IgG and relies on the pinocytic nature of insect PCs, while the second employs LysoTracker Red and relies on the ability of this stain to label cells with high acidic, and presumably lysosomal, content.

Co-staining of PCs and the abdominal musculature showed that PCs are binucleated cells that flank the mosquito heart (Figure 2C). PCs occur along the length of the heart, with large gaps present between the diamond shaped alary muscles that tether the heart to the cuticular epidermis, and small gaps present slightly posterior of the anterior-posterior midline of the alary muscles. Based on flow experiments (movie S1) as well as our previously published work on the structural mechanics of the heart [3], we conclude that the smaller gaps flank the heart's ostia, and thus, in this study we refer to these areas as the periostial regions (Figure 1).

Staining of PCs after infection with GFP-expressing *Escherichia coli* showed that pathogens are not phagocytosed by PCs. Instead, pathogens accumulate at the periostial regions between the four PCs that flank the ostia (Figure 2D). Further examination of LysoTracker Red-stained dorsal abdomens revealed that the accumulated bacteria are inside cells with nuclear and cellular diameters that are considerably smaller than that of PCs, and that these phagocytic cells are similar in size to the phagocytic subpopulation of circulating hemocytes (granulocytes) (Figure 2E). Thus, these data show that pathogen accumulation in the periostial regions is due to an immune response that is not mediated by PCs, and suggest that the phagocytic cells are likely hemocytes that bind the alary muscles and the outer surface of the heart at the location of the ostia.

### CM-DiI selectively stains hemocytes in vivo

**Figure 3:**
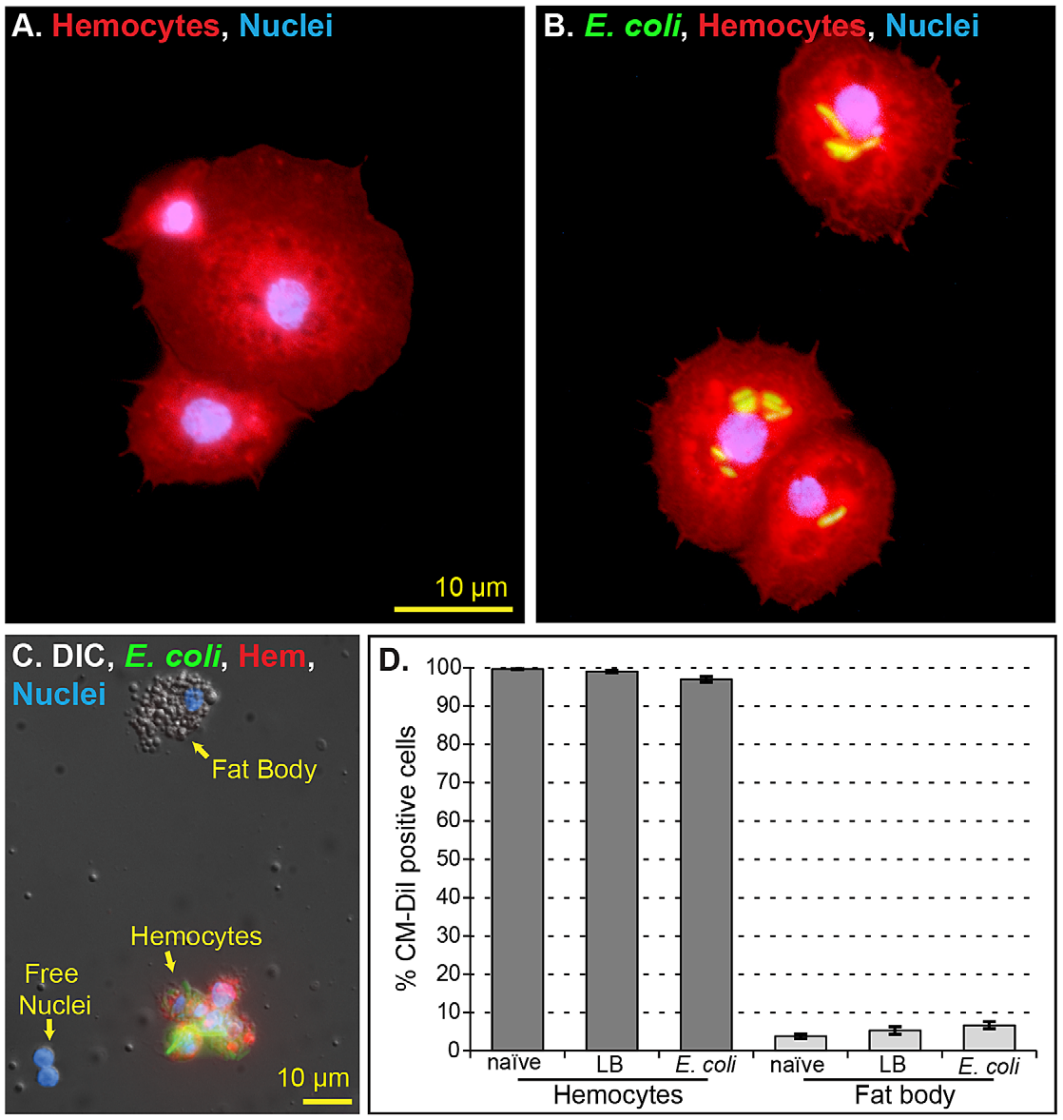
CM-DiI selectively stains hemocytes in vivo. (A–B) Perfused hemocytes from mosquitoes injected with CM-DiI (red) and Hoechst 33342 (blue; nuclear stain). CM-DiI stains individual hemocytes (B) and hemocyte aggregates (A) from both naïve (A) and *E. coli*-infected (B) mosquitoes. (C) Bright field and fluorescence overlay of hemocytes, fat body and free nuclei collected by perfusion. Only hemocytes stain with CM-DiI. (D) Quantitative analysis of in vivo CM-DiI staining in perfused cells from naïve, injured (LB) and *E. coli*-infected mosquitoes. Greater than 97% of hemocytes stain with CM-DiI, fewer than 5% of fat body cells stain with CM-DiI, and free nuclei do not stain with CM-DiI. Columns, mean; bars, standard error of the mean.

To determine whether the phagocytic cells identified in the periostial regions are hemocytes, we developed a novel in vivo hemocyte-staining method using the dye chloromethylbenzamido-1,1′-dioctadecyl-3,3,3′,3′-tetramethylindocarbocyanine-perchlorate (CM-DiI). Intrathoracic injection of CM-DiI into live mosquitoes, followed by hemolymph perfusion and ex vivo analysis of cell staining efficiencies showed that CM-DiI stains greater than 97% of the hemocytes collected from naïve (99%), injured (sterile injection with LB broth; 99%) and *E. coli* infected (97%) mosquitoes (Figure 3A–D). Quantitative co-localization of CM-DiI staining and the phagocytosis of GFP-*E. coli* by circulating hemocytes yielded a Mander's overlap of 70%, illustrating the high hemocyte-staining efficacy of CM-DiI. Further qualitative analyses suggested that the slightly lower hemocyte-staining efficiency seen in *E. coli* infected mosquitoes is due to lower incorporation of CM-DiI in hemocytes that have phagocytosed extremely large numbers of bacteria.

In addition to hemocytes, hemolymph perfusion results in the collection of small amounts of fat body as well as free nuclei from lysed cells [17], [18]. Visual analysis of all cellular components collected by perfusion revealed that CM-DiI only stains hemocytes (Figure 3C–D), and analysis of carcasses and other tissues from CM-DiI injected mosquitoes conclusively showed that cells other than hemocytes do not incorporate CM-DiI.

### Hemocytes form periostial immune foci, which occur in close proximity to the pericardial cells

**Figure 4:**
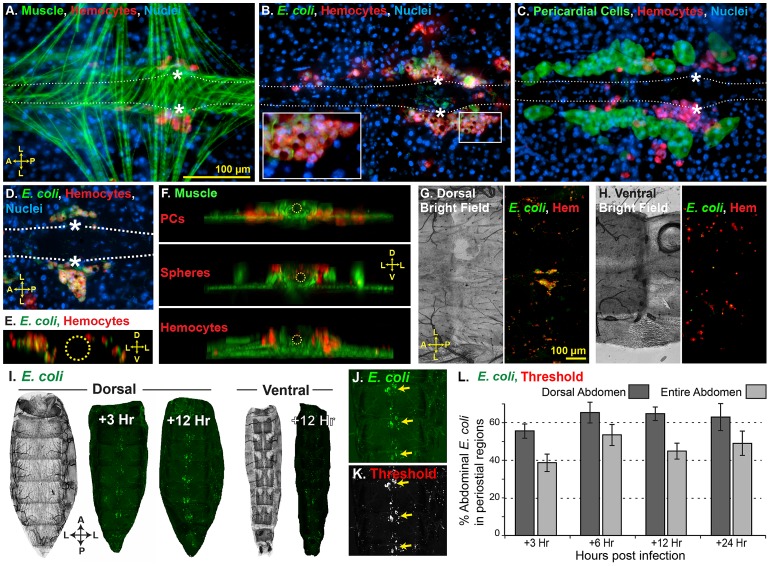
Hemocytes form periostial phagocytic foci, which occur in close proximity to the pericardial cells (PCs). (A) Heart musculature (phalloidin; green) and hemocytes (CM-DiI; red) 24 h following infection with non-fluorescent *E. coli*, showing hemocytes at the periostial regions. (B) Periostial hemocytes (red) 24 h after GFP-*E. coli* (green) infection, showing phagocytosis and melanization (dark spots) reactions. (C) Pericardial cells (488 nm-IgG; green) and hemocytes (red) following infection with non-fluorescent *E. coli*, showing that PCs and periostial hemocytes are structurally distinct. (D–E) Fluorescence overlay (D) and 3D volume view (E; dotted circle marks the lumen of the heart) of periostial phagocytic foci, demonstrating the co-localization of phagocytosis and CM-DiI stained hemocytes in all three dimensions. (F) 3D-rendered volume views illustrating the spatial separation of periostial hemocytes and PCs. The point of view is looking through the lumen of the vessel (yellow circle), with the heart located dorsally and the alary muscles extending laterally in the ventral portion of the image. The PCs (red; 568 nm-IgG) occur within the fan-like alary muscles (green horizontal line; phalloidin), while phagocytosis (red microspheres) and hemocytes (red; CM-DiI) occur toward the dorsal edge of the heart. (G–H) Dorsal (G) and ventral (H) views of one abdominal segment (centered around an abdominal suture) showing that periostial foci are the major hemocyte aggregates within the mosquito's abdomen. (I) *E. coli* uptake in the dorsal and ventral abdomen at 3 and 12 h post-infection. Phagocytosis levels in the dorsal abdomen increase between 3 and 12 h post-infection. (J–L) Quantification of *E. coli* fluorescence in the abdomen. Images of the dorsal and ventral abdomen were acquired (portion of an image from the dorsal abdomen shown in J), thresholded (K), and the percentage of *E. coli* fluorescence in the periostial regions relative to the dorsal abdomen or the entire abdomen (dorsal+ventral) was calculated (L). Between 6 and 24 h post-infection, sum intensity of periostial *E. coli* is ≥60% and ≥40% of the dorsal and entire abdominal *E. coli*, respectively. Bars represent the standard error of the mean. D, dorsal; V, ventral; A, anterior; P, posterior; L, lateral; asterisks, location of the ostia; dotted lines; outline of the heart. In panels A–D, Hoechst 33342 (blue) was used as a nuclear stain.

With the knowledge that a major cellular immune response occurs in the periostial regions, we then aimed to determine whether this immune response is mediated by hemocytes. Complementary hemocyte, PC, and muscle staining confirmed that this indeed is the case. Co-labeling of hemocytes and muscle showed that hemocytes laterally flank the heart at the periostial regions in both naïve and *E. coli*-infected mosquitoes (Figure 4A). Infection with GFP-*E. coli*, followed by CM-DiI injection 24 h later, revealed strong spatial overlap between GFP-fluorescence and hemocytes (Figure 4B), and suggested that hundreds of hemocytes phagocytose pathogens in the periostial regions during an active infection. Then, examination of abdomens from mosquitoes infected with non-fluorescent *E. coli* confirmed that PCs and hemocytes are two distinct cell populations, and that hemocytes occur in aggregates positioned between the 4 PCs that flank each ostial pair (Figure 4C). Finally, comparisons of cellular and nuclear diameters confirmed that hemocytes are identical in size to (1) the phagocytic cells previously detected using LysoTracker Red staining (Figure 2E), and (2) the circulating population of granulocytes (Figure 3A–C).

Because immune processes within the mosquito hemocoel occur in three-dimensional space, we performed a series of dual labeling experiments to elucidate the three-dimensional relationship between bacterial aggregation and the periostial hemocytes, and the relationship between hemocytes, PCs and the heart. Analysis of deconvolved and volume rendered Z-stacks revealed that aggregated periostial hemocytes and *E. coli* overlap in all three dimensions, supporting the phagocytic activity of heart-associated hemocytes (Figure 4D–E). Then, similar experiments where heart muscle was labeled along with PCs, phagocytosis foci or hemocytes showed that PCs intertwine with the alary muscles while phagocytosis foci and periostial hemocytes are located dorsal of the alary muscles and in the vicinity of the ostia (Figure 4F).

Finally, because sessile hemocytes occur elsewhere in the mosquito, we examined whether hemocytes or bacteria preferentially aggregate elsewhere in the abdomen. We found that, throughout the abdomen, the periostial regions are the major location of hemocyte aggregation following infection (Figure 4G–H), as well as the major location of *E. coli* sequestration (Figure 4I–L). Quantitative analysis of the dorsal abdomen showed that between 6 and 24 h post-infection, ≥62% of *E. coli* aggregation, as measured by GFP fluorescence, is confined to the periostial regions (Figure 4J–L). When the analysis was repeated for the entire abdomen (dorsal+ventral), ≥42% of the aggregated *E. coli* was confined to the periostial regions. Taken altogether, these data describe a novel mosquito immune response, where hemocytes in the periostial regions of the heart phagocytose and degrade pathogens.

### Infection induces the migration of hemocytes to the periostial regions

**Figure 5:**
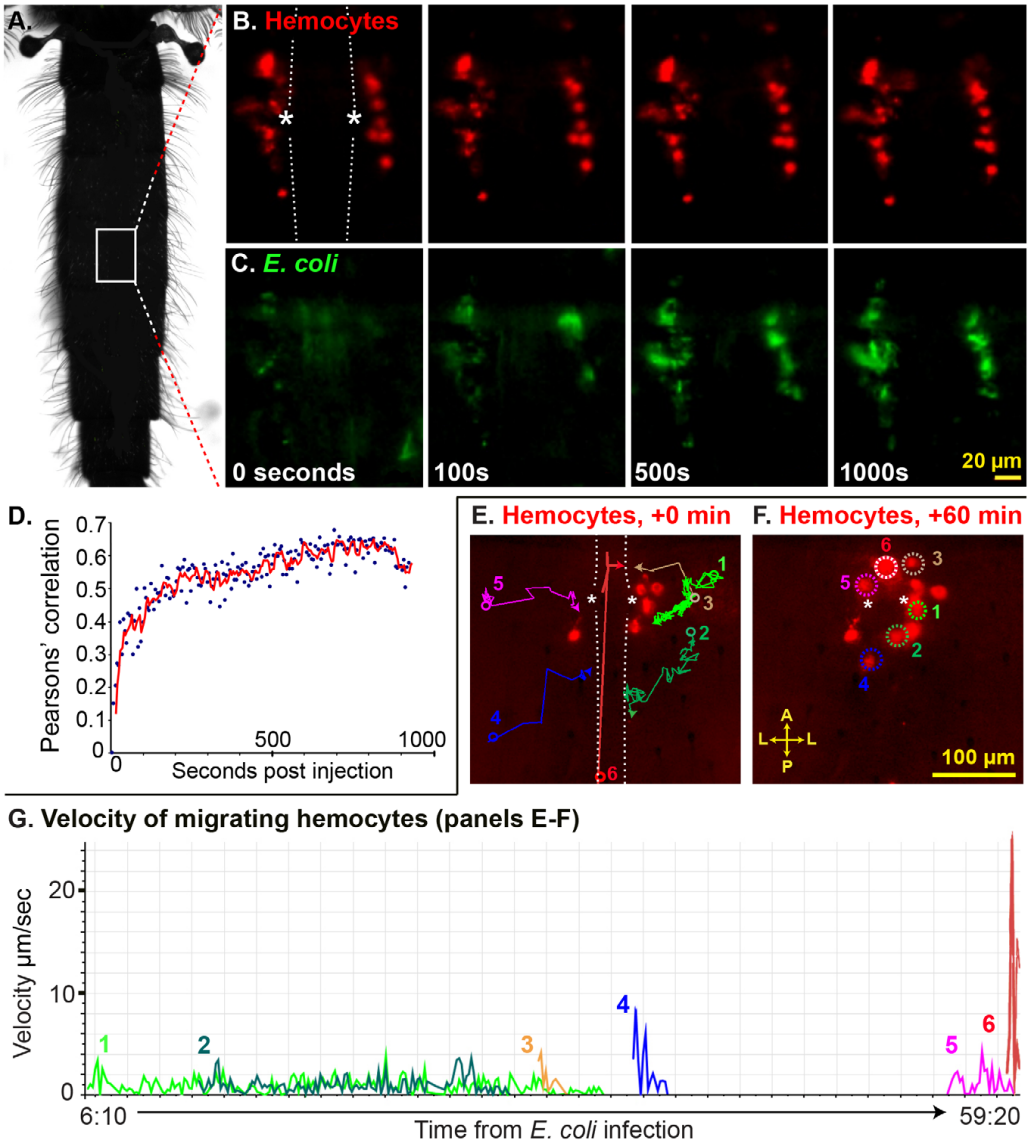
Periostial hemocytes rapidly phagocytose pathogens and infection induces the recruitment of additional periostial hemocytes. (A) Top-down view of a mosquito, outlining the region observed in panels B, C, E and F. (B–C) Time-lapse images of a periostial region of a mosquito whose hemocytes had been labeled with CM-DiI prior to infection (Movie S1). The heart is outlined by dotted lines and the asterisks denote the ostia. During the first 1,000 sec post-infection with *E. coli* little hemocyte (red) movement is observed (B), but phagocytosis of *E. coli* (green) by resident periostial hemocytes begins within seconds of infection (C). (D) Pearson's correlation coefficient analysis of Movie S1 (B–C), quantitatively showing the rapid co-localization of hemocytes and *E. coli*. (E–F) Time-lapse images showing the migration of hemocytes (red) to the periostial regions during the first hour post-infection with *E. coli* (Movie S2). At the time of infection, 6 CM-DiI stained hemocytes flank the heart (E), and during the first hour of infection at least 6 additional hemocytes attach at the periostial region (F; dotted circles). The movement of each migrating hemocyte is shown in panel E using colored lines, with the circles marking the points of first observance and the arrows marking the points of attachment. (G) Velocity and acceleration of the hemocytes tracked in panels E and F as they migrate to the periostial regions (Movie S2), showing that hemocytes glide across the alary muscles at a median velocity of 1.2 µm/sec. A, anterior; P, posterior; L, lateral.

Because periostial hemocytes appeared to represent a substantial proportion of the total number of hemocytes present in mosquitoes [17], [19]–[23], and because their positioning suggested a strong interaction between the mosquito circulatory and immune systems, we then investigated whether hemocytes are recruited to the periostial regions during the course of an infection. Intravital video imaging of periostial hemocytes during the first 15 min post-infection revealed that resident sessile periostial hemocytes (basal population of hemocytes always present at the periostial regions) phagocytose *E. coli* within seconds of their injection into the hemocoel (Figure 5A–C; Movie S1). Pearson's correlation coefficient measurements of Movie S1 quantitatively proved this, as the fluorescence overlap of periostial hemocytes and *E. coli* increased within the first few frames of the video and began to plateau at a correlation near 60% by 3 min post-infection (Figure 5D). Longer video recordings confirmed the high phagocytic activity of resident periostial hemocytes, and also showed that the number of hemocytes in the periostial regions increases during the course of an infection (Figure 5E–G; Movie S2). Specifically, during the first hour of infection with *E. coli* (OD_600_ = 4), the number of hemocytes in the periostial regions roughly doubles. This increase is due to the movement of hemocytes into the periostial regions and not hemocyte replication at the pericardium. As seen in Movie S2, some hemocytes flowing into the pericardium adhere to the heart-associated musculature. These hemocytes then slowly glide into the periostial regions, where they settle and phagocytose pathogens. While hemocytes exist both in circulation and attached to tissues (sessile), the relatively low number of sessile hemocytes observed in non-periostial areas of the dorsal cuticular epithelium, together with the relatively rapid arrival of hemocytes into the pericardium suggests that the recruited cells originate from the circulating hemocyte population. The molecular trigger for their arrival is unclear, but we hypothesize that hemolymph flow brings circulating hemocytes to the pericardium, where they bind the alary muscles and then slowly undergo a directed migration into the periostial regions. This migration is facilitated by hemolymph flow but is not exclusively driven by it; the median velocity of the hemocytes tracked in Movie S2 was 1.2 µm/sec (Figure 5G), which is several orders of magnitude slower than the 200–1,000 µm/sec and ∼8,000 µm/sec hemolymph flow velocities in the periostial regions and heart lumen, respectively [3]. Overall, while the vast majority of hemocytes migrate to the periostial regions as individual cells, a minor proportion of hemocytes in a minority of mosquitoes arrives at the pericardium in small cellular aggregates. Many single or aggregated migrating hemocytes contain phagocytosed *E. coli* prior to entering the pericardial space, indicating that they have been immune activated elsewhere in the mosquito.

**Figure 6:**
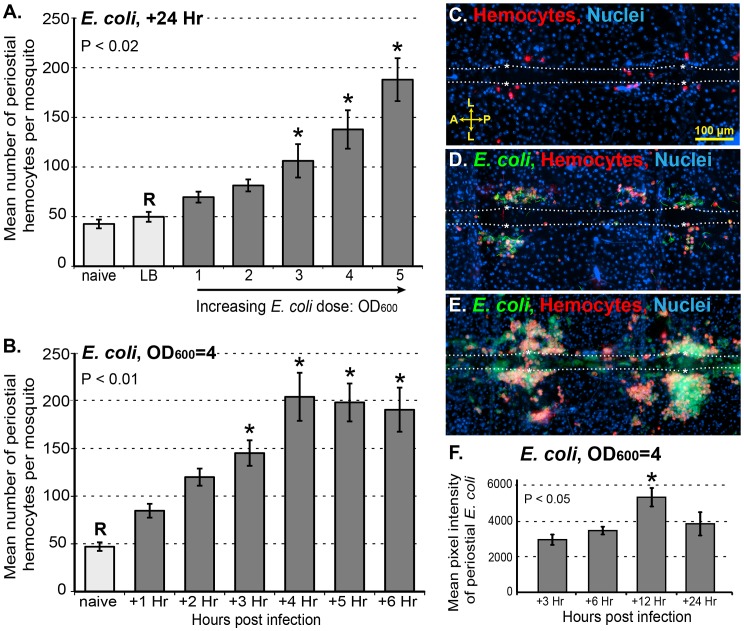
Periostial hemocyte aggregation occurs in a dose and time dependent manner, and leads to bacterial death. (A) Periostial hemocytes numbers in naïve mosquitoes, injured mosquitoes (LB), and mosquitoes infected for 24 h with *E. coli* (OD1–OD5). Resident periostial hemocytes are present in naïve mosquitoes and injury does not trigger hemocyte recruitment, but infection triggers hemocyte migration to the periostial regions in a dose-dependent manner. (B) Dynamics of periostial hemocyte numbers in naïve and *E. coli* (OD4)-infected mosquitoes. Following high-intensity infection, hemocyte recruitment to the periostial regions is complete by 4 h post-infection. Columns mark the mean and bars denote the standard error of the mean. P-values result from one-way ANOVA, and asterisks denote treatment groups that are significantly different from the reference group (R; Tukey's test). (C–E) Resident periostial hemocytes (red) in naïve mosquitoes (C), and both small (D) and large (E) periostial hemocyte aggregates in GFP-*E. coli* (green)-infected mosquitoes. (F) Average GFP-*E. coli* intensity in the periostial regions at 3, 6, 12 and 24 h following infection. *E. coli* intensity peaks at 12 h post-infection and then recedes. Columns mark the mean and bars denote the standard error of the mean. P-value results from one-way ANOVA, and asterisk denotes the treatment group that is significantly different all others (R; Tukey's test). A, anterior; P, posterior; L, lateral; asterisk, location of the ostia; dotted lines, outline of the heart. Hoechst 33342 (blue) was used as a nuclear stain.

To further elucidate the process of periostial hemocyte aggregation, naïve mosquitoes, mosquitoes that had been injured 24 h earlier, and mosquitoes that had been infected with various doses of GFP-*E. coli* for 24 h were injected with CM-DiI and the number of periostial hemocytes were counted. On average, 5-day-old naïve mosquitoes contain 43 periostial hemocytes, distributed among the 6 periostial regions of the abdomen (Figure 6A, C). Injury does not result in an increase in the number of periostial hemocytes, but infection with large numbers of *E. coli* for 24 h leads to a >4-fold increase in the number of periostial hemocytes (Figure 6A). Hemocyte aggregation in the periostial regions is induced in an infection dose-dependent manner (Figure 6A), with the increases in mean number of periostial hemocytes per mosquito being nearly linear for *E. coli* infection intensities of OD_600_ = 1 through OD_600_ = 5 (R^2^ = 0.92; means of 69, 89, 106, 138, and 188, respectively). Quantitative analysis of periostial hemocyte numbers during the course of an infection revealed that hemocyte numbers in the periostial regions approximately double within the first hour, and plateau at 4 h post infection (Figure 6B). Depending on the bacterial dose and the time following infection, periostial hemocyte aggregates vary from small and dispersed groups of hemocytes to expansive and contiguous aggregates of hemocytes (Figure 6C–E). Infected mosquitoes at times contain more than 300 periostial hemocytes (Figure 6E), even when counting is done using our conservative protocol, which favors the exclusion of a small number of hemocytes rather than the inclusion of non-hemocytes. Finally, quantitative analysis of *E. coli* fluorescence in the periostial regions showed that the level of viable (fluorescent) bacteria in the periostial regions steadily increases until 12 h post-infection and then begins to decline (Figure 6F). This loss of GFP-fluorescence in the periostial regions strongly suggests that bacterial killing and degradation is dynamically occurring in these regions.

**Figure 7:**
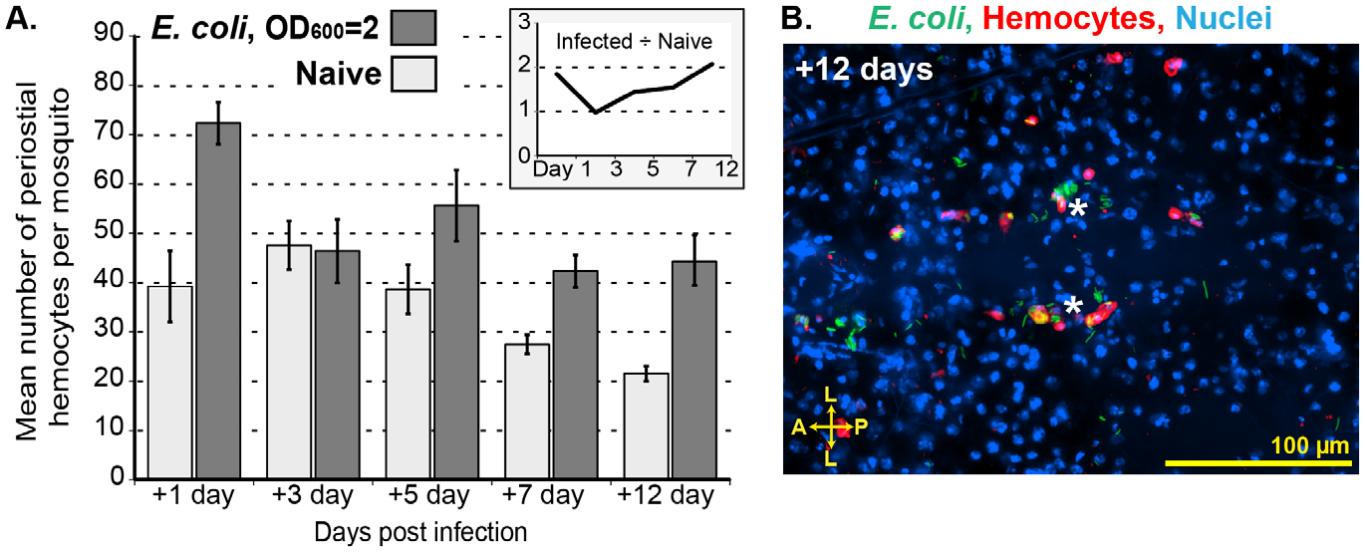
Periostial hemocyte aggregates begin to disperse several days after infection. (A) Periostial hemocyte numbers in naïve mosquitoes and mosquitoes infected with low levels of *E. coli* (OD2) for 1, 3, 5, 7, and 12 days. Columns mark the mean and bars denote the standard error of the mean. The numbers of hemocytes in infected mosquitoes returns to pre-infection levels by 3 days post-infection, but are maintained at higher levels relative to naïve mosquitoes for the lifetime of the mosquito. (A, inset) Ratio of hemocytes in infected vs. naïve mosquitoes at different times post-treatment. (B) *E. coli* (green) persists at 12 days post infection, with hemocytes (red; CM-DiI) remaining immunologically active in the periostial regions. A, anterior; P, posterior; L, lateral; asterisk, location of the ostia; dotted lines, outline of the heart. Hoechst 33342 (blue) was used as a nuclear stain.

While infection induces the migration of hemocytes to the periostial regions, their numbers decrease once the acute stage of infection has passed. Infection of mosquitoes with large numbers of *E. coli* (OD_600_ = 4) revealed that hemocytes are recruited to the periostial regions within the first 24 h post-infection, but that the number of periostial hemocytes decreases by day 3 post-infection (not shown). Infection of mosquitoes with fewer *E. coli* (OD_600_ = 2) revealed a similar trend: the number of periostial hemocytes decreases significantly by 3 days post-infection, but remains elevated for the lifetime of the mosquito, relative to similarly aged naïve controls (Figure 7A). All mosquitoes assayed at 12 days post challenge had live *E. coli* in their hemocoels (and periostial regions; Figure 7B), and the amount of melanin deposition in the periostial regions also increased as mosquitoes aged. Thus, the maintenance of elevated numbers of periostial hemocytes may be due to the continued need of cellular antimicrobial activity, as mosquitoes appear to be incapable of completely clearing an *E. coli* bacterial infection [24], [25]. Finally, based on the number of circulating hemocytes [17], [19]–[23], the periostial hemocyte population represents between 10% and 25% of the total hemocyte population post-infection, which highlights the importance of pathogen sequestration in these areas of high hemolymph flow.

### Soluble immune elicitors induce periostial hemocyte aggregation

**Figure 8:**
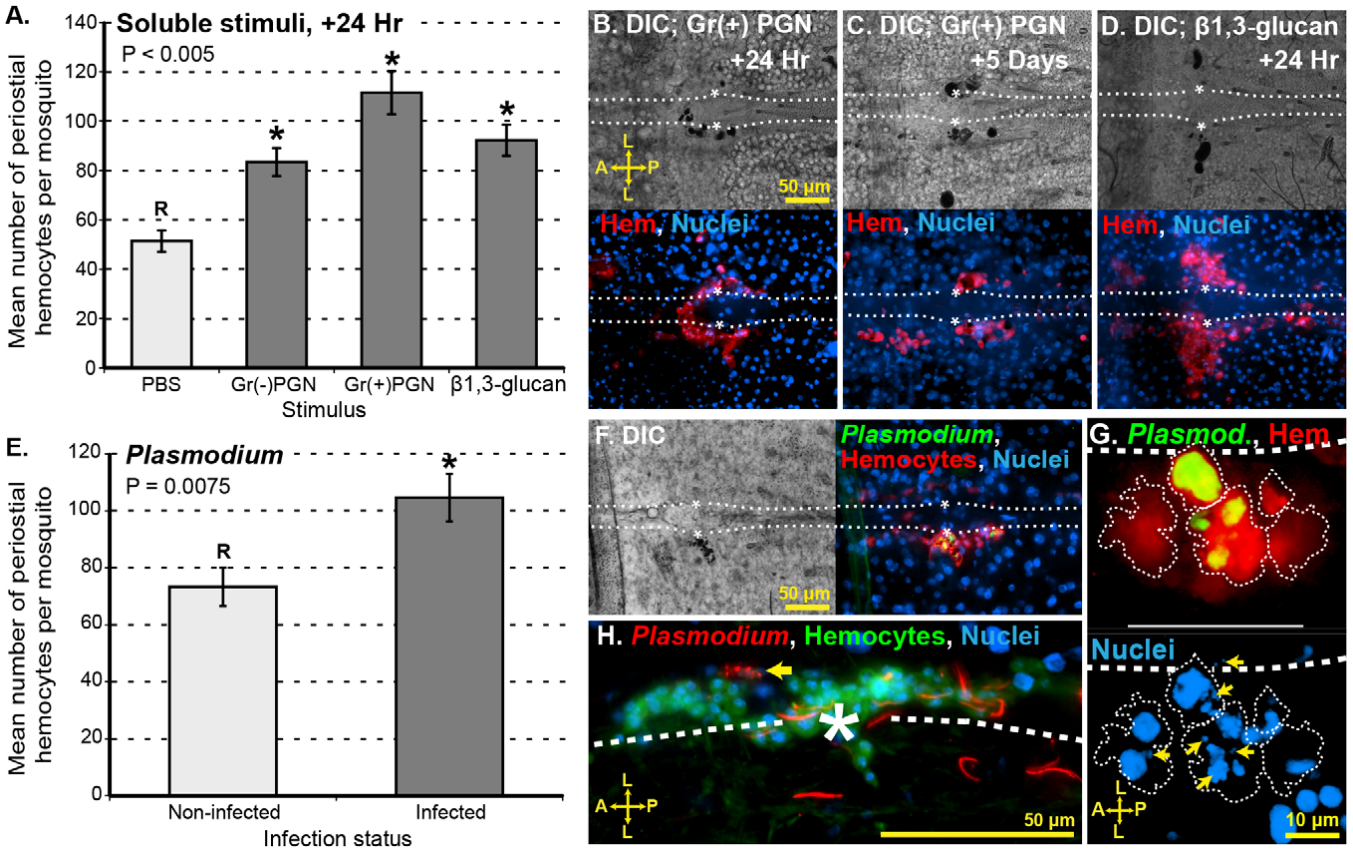
Soluble immune elicitors and *Plasmodium berghei* infection induce periostial hemocyte aggregation. (A) Hemocyte numbers in the periostial regions following treatment with soluble immune elicitors. Columns mark the mean and bars denote the standard error of the mean. P-value results from one-way ANOVA, and asterisks denote treatment groups that are significantly different from the reference group (R; Tukey's test). Peptidoglycan (PGN) from both Gram (−) and Gram (+) bacteria, as well as β-1,3-glucan, elicit the migration of hemocytes to the periostial regions. (B–D) DIC and fluorescence images of a periostial region at 24 h and 5 days after Gram (+) peptidoglycan injection (B–C) or 24 h after β-1,3-glucan injection (D). Large hemocyte aggregates (CM-DiI; red) and melanin deposits (black) are observed. (E) Periostial hemocyte numbers 20 days after mosquitoes received a normal (non-infected) or *Plasmodium berghei*-infected blood meal. Columns mark the mean, bars denote the standard error of the mean, and the P-value results from an unpaired t-test. *Plasmodium* sporozoite migration induces hemocyte aggregation in the periostial regions. (F) DIC and fluorescence images of a periostial region at 20 days following infection with *Plasmodium berghei*. Signs of melanization (black) were sometimes present and overlap was often seen between periostial hemocytes (CM-DiI; red) and green puncta, which represent the remnants of destroyed sporozoites (PbGFP_CON_; green). (G) Hemocyte (CM-DiI; red; cell boundaries are outlined) and *Plasmodium* (PbGFP_CON_; green) fluorescence overlay (top), showing the phagocytosis of multiple sporozoites by periostial hemocytes. Hoechst 33342 fluorescence channel (bottom) shows that the nuclei of *Plasmodium* sporozoites (arrows) are contained within periostial hemocytes. (H) Interactions between *P. berghei* sporozoites (Pb RedStar; red) and periostial hemocytes (CM-DiO, a CM-DiI analog; green), with arrow pointing to hemocyte-mediated *Plasmodium* fragmentation. A, anterior; P, posterior; L, lateral; asterisk, location of the ostia; dotted lines, outline of the heart. Hoechst 33342 (blue) was used as a nuclear stain.

Phagocytosis of 1 µm diameter microspheres triggers the recruitment of hemocytes to the periostial regions (Figure 2A–B), suggesting that immune activation via phagocytosis pathways induces hemocyte migration to the pericardial space. We tested whether several soluble immune elicitors also induce hemocyte aggregation in the periostial regions. Being solubilized, these microbial components are outside of the size range that induces phagocytosis. Examination of dorsal abdomens 24 h after injection with peptidoglycan and β-1,3-glucan revealed that soluble immune elicitors also induce the aggregation of hemocytes in the periostial regions (Figure 8A–D). The aggregation response following treatment with PGN and β-1,3-glucan was lower than following infection with live *E. coli* (Figure 6A), but this reduced response could be related to the doses used (see Figure 6A) and may not be an indication that soluble immune elicitors are less (or more) capable of inducing periostial hemocyte aggregation. Melanization in the periostial regions was prevalent following injection with peptidoglycan and β-1,3-glucan (Figure 8B–D), and this melanization response was considerably stronger than what was observed following infection with *E. coli*. Finally, while periostial hemocyte aggregation remained elevated several days following injection of soluble elicitors, hemocytes at these later time-points could not be accurately counted because of the extensive melanization response. Taken altogether, these data suggest that periostial hemocyte aggregation is a basal immune response that is induced by a broad range of immune stimuli.

### *Plasmodium* infection induces periostial hemocyte aggregation

Mosquitoes are vectors of disease-causing pathogens. Among the most important mosquito-borne pathogens are *Plasmodium* parasites, which are the etiological agents of malaria. *Plasmodium* infection represents a complex and long-term immune stimulus for mosquitoes [11], [26], [27], and we have previously observed *Plasmodium* sporozoites near the heart's ostia [11]. For these reasons, and because *Plasmodium* infection occurs without breaching the outer cuticle, we analyzed whether *Plasmodium* infection induces periostial hemocyte aggregation. Examination of the periostial regions of mosquitoes that had received a normal blood meal and mosquitoes that had received a *Plasmodium*-infected blood meal revealed that the process of sporozoite migration to the salivary glands induces the aggregation of hemocytes in the periostial regions: mean numbers of periostial hemocytes increased from 72 in the non-infected group to 106 in the infected group (Figure 8E). Moreover, periostial hemocytes phagocytosed *Plasmodium* sporozoites, and a minority of these sporozoites were also melanized (Figure 8F–H). In some cases, multiple *Plasmodium* nuclei were observed within an individual hemocyte (Figure 8G). Some of these sporozoites were fragmented and exhibited varying levels of fluorescence intensity, which is indicative of death or dying (Figure 8G–H). Overall, these data show that *Plasmodium* infection induces the recruitment of hemocytes to the periostial regions, and suggest that the cellular immune response against *Plasmodium* may be stronger than what was reported in an earlier study [11] that assayed circulating hemocytes alone.

## Discussion

Based largely on practical constraints, the insect immune and circulatory systems have been conceptually divided into discrete elements, and the immune system further dissected into cellular and humoral components [28]. However, these entities are physiologically interrelated and have apparently evolved in integral association since the beginning of animal evolution [28]–[32]. The cellular immune response remains only partially understood in mosquitoes as well as in other adult insects [5], [7]. Likewise, interactions between major circulatory elements and immune cells are virtually unknown and have received little attention. Using methods we previously developed for the study of hemolymph circulation [2], [3], along with novel techniques for the in vivo investigation of hemocyte biology, we analyzed the cellular immune response in the pericardial region of the malaria mosquito, *Anopheles gambiae*. We discovered that during an active infection, hemocytes migrate to the periostial regions, where they form a major component of the cellular immune response. Exemplifying the interrelationship of cellular immunity and circulatory processes, periostial hemocytes form phagocytic foci in regions of high hemolymph flow, which are also in the direct vicinity of the mosquito's major nephrocytes, the pericardial cells. Together, the data presented herein describe the formation of a novel immune tissue in mosquitoes. Because previous mosquito studies did not recognize sessile hemocyte aggregations as a major player in immunity, they failed to examine a large proportion of the cellular immune response, and thus, underestimated the relative contribution of hemocytes in anti-pathogen responses.

Using a correlative imaging approach, we scrutinized what appeared to be major phagocytic foci forming in the periostial regions of the mosquito heart. We previously reported that these foci form in response to infection, but their cellular composition and their functional role remained unknown [11]. Similar foci form in *Drosophila*, although they have never been directly studied [10], [33]. Here, we dispel the notion that phagocytosis on the surface of the heart is due to the activity of PCs. This finding was not entirely surprising, as in two different insect orders the PCs have been shown to be surrounded by a basement membrane [32], [34], [35], and the presence of this physical barrier should impede the direct phagocytosis of invading pathogens. Instead, we report that immune foci on the surface of the heart are composed of periostial hemocytes that rapidly and efficiently phagocytose pathogens. Rapid phagocytosis is believed to be an essential immune process, which culminates in pathogen death and the production of humoral immune components [36], [37]. The large number of periostial hemocytes present in infected mosquitoes, when compared to the total number of circulating hemocytes [17], [19]–[23], suggests that this response involves between 10% and 25% of the hemocytes present in mosquitoes, thus highlighting the importance of pathogen sequestration in areas of high hemolymph flow. Finally, because nodulation and cellular encapsulation do not occur in mosquitoes [5], [7], periostial foci formation is the primary hemocyte aggregation immune response in the culicid lineage.

Periostial immune aggregates are composed of a mixture of resident hemocytes and circulating hemocytes that settle in the pericardial regions in response to infection. Given that bacterial infection induces an increase in the number of circulating hemocytes in *An. gambiae* [21], and that *Aedes aegypti* hemocytes can replicate in response to various stimuli [19], [20], we hypothesize that some of the migrating hemocytes seen in this study are the products of circulating hemocyte replication in response to infection. While the origin of periostial hemocytes seems clear, the molecular trigger that induces hemocyte aggregation in the periostial regions is unknown. The finding that hemocyte recruitment is induced by bacteria, *Plasmodium*, carboxylate modified latex microspheres and soluble immune elicitors suggests that multiple pathways of immune activation can induce periostial hemocyte aggregation. Studies in other insects have identified several molecular components involved in sessile hemocyte aggregation and release. For example, Noduler mediates hemocyte aggregation in Lepidoptera [38], and multiple pathways mediate hemocyte proliferation and adhesion in *Drosophila* [39]. However, a great amount of genomic divergence is seen in insect immune genes [40], [41], and thus, alternate pathways may be involved in mosquitoes.

What is perhaps most interesting about periostial hemocyte aggregates is their location. In dipterans, the cardiac ostia are the major incurrent valves for hemolymph entry into the heart [2], [3], [42]. Thus, hemocyte aggregation in these regions greatly increases the likelihood that hemocytes encounter circulating pathogens, and that toxic products produced during pathogen breakdown are either immediately diluted as they are swept into the rapidly flowing hemolymph or are captured by the nephrocytic PCs that flank the heart. PCs filter proteins and colloids from the hemolymph [32], and thus, it is likely that the proximity of the PCs to the periostial hemocytes is essential for the quick absorption of pathogen breakdown products. We speculate that, during the course of arthropod evolution, periostial hemocytes and PCs adapted to their current locations because of their proximity to each other in an area of high hemolymph flow.

Data from the first hour post-infection showed that circulating hemocytes bind the alary or cardiac musculature within 100 µm of the ostia and then glide into the periostial regions at velocities that are orders of magnitude slower than that of the surrounding hemolymph flow. The molecular mechanism for this process was not a focus of this study, but the movement of insect hemocytes is known to be controlled by a number of different molecular pathways, and to be governed by processes such as adhesive capture and chemotaxis [43]–[47]. The process observed here is likely a variant of adhesive capture, a process described in *Drosophila* where injury induces the capture of hemocytes at epidermal wound sites [43]. In agreement with the process of adhesive capture is our observation that mosquito hemocytes that reach the periostial regions originate from the circulating pool of hemocytes; video analyses show the binding of hemocytes to the alary and cardiac musculature and not the gliding of sessile hemocytes across extended distances. However, in contrast with adhesive capture in *Drosophila*, where individual hemocytes arrive at injury sites and do not disperse or migrate [43], mosquito hemocytes bind within the general vicinity of the ostia and then move into their final point of attachment at velocities considerably slower than that of hemolymph flow. This spatially directed gliding process is likely mediated by shear-flow dynamics, a process that has been shown to drive cell migration toward areas of high flow in phylogenetically diverse organisms [48], [49]. This process is also reminiscent of the early stages of vertebrate leukocyte extravasation in response to inflammation, where activated endothelial cells produce factors that capture circulating leukocytes, at which point the leukocytes roll in the direction of flow, and then undergo diapedesis [50], [51]. Finally, on rare occasions hemocytes arrive into the pericardial regions as small aggregates. Thus, there remains the possibility that hemocyte aggregation in circulation, or increases in hemocyte size post-infection [25], [37], [52], are physical mechanisms for an evolutionarily pragmatic response to infection.

In a manner comparable to what we describe in mosquitoes, the macrophages of many lower vertebrates aggregate in areas of high blood flow in response to infection [53], [54]. These aggregates assemble in the spleen and liver, where they concentrate the destruction and recycling of exogenous and endogenous material. Likewise, the tissue macrophages of higher vertebrates (e.g., stellate cells and Kupffer cells) are believed to have originated from the same cellular ancestors as the phagocytes found in all animals, and are also commonly located in areas of high blood flow [55]. It seems parsimonious to speculate that such phylogenetically disparate phagocyte aggregation responses are entirely based on functional analogy. However, mounting evidence suggests that these aggregation responses rely on conserved molecular and physiological components that were present in an ancient bilaterian [29]–[32], [55], [56]. From a physiological perspective, we believe the data presented here exemplifies the taxonomically widespread importance of evolutionary constraints imposed on the cellular branch of the immune system as a consequence of its long history of evolution in close association with the circulatory system.

Although it is known that most *Plasmodium* sporozoites rapidly die during their migration through the mosquito hemocoel [11], the specific interactions between sporozoites and hemocytes remain largely unknown. Earlier reports showed that phagocytosis of *Plasmodium* by hemocytes occasionally occurs [11], [37]. Our data show the in vivo interaction between *Plasmodium* and hemocytes, along with the first evidence of a systemic cellular immune response to late-stage malaria infection (increase in periostial hemocyte numbers). While the large number of sporozoites released by each oocyst makes it unlikely that phagocytosis is the primary component of the anti-*Plasmodium* response in the hemocoel, increases in melanization and periostial hemocyte aggregation suggest that hemocyte activation leads to the production of humoral factors that target *Plasmodium* via lytic and melanization pathways. Evidence from others supports this idea, as *Plasmodium* development in mosquitoes induces the transcriptional regulation of immune genes in hemocytes [57], [58], and our data on melanin deposition near hemocyte-*Plasmodium* interactions are in agreement with studies on the anti-sporozoite response [37], [59].

In most insects, one problem foiling hemocyte research is that no effective means of specifically staining hemocytes in vivo exists [17], [58]. As part of this investigation, we developed a CM-DiI based method that fluorescently labels hemocytes in vivo. CM-DiI is a lipophilic probe with high affinity to plasma membranes. While this probe stains all cells grown in culture, it only stains hemocytes when injected into mosquitoes. Several lines of evidence support the specificity and efficiency of CM-DiI hemocyte labeling. First, CM-DiI stains virtually all circulating hemocytes and also stains cells attached to tissues in a random pattern (sessile hemocytes). Second, CM-DiI stains cells that fit the morphological description of hemocytes given by previous authors [17], [18], [37]. Finally, the vast majority of cells that stain with CM-DiI exhibit the characteristic phagocytic signature of mosquito granulocytes. Although the mechanism by which CM-DiI specifically stains hemocytes remains unknown, our data suggest that CM-DiI is unable to cross the basal lamina surrounding internal tissues, and that it initially stains hemocytes by binding their membranes and then becoming subsequently phagocytosed. Evidence supporting this mechanism of labeling includes: (1) co-injection of formaldehyde along with CM-DiI eliminates hemocyte specificity and all tissues become labeled; (2) incubation of CM-DiI injected mosquitoes in a solution containing a detergent releases the hemocyte-captured CM-DiI and leads to the staining of other tissues; (3) injection of carbon particles prior to CM-DiI treatment blocks hemocyte staining; (4) CM-DiI staining appears most brightly as puncta within the hemocytes but over time spreads over the entire cell membrane; and (5) within minutes of mixture with PBS, CM-DiI precipitates out of solution and loses its hemocyte staining efficacy. Taken altogether, this suggests that CM-DiI could be categorized as a functional marker that stains only hemocoelic phagocytes in vivo. Techniques based on similar principles have been used for the study of macrophage biology in mammals [60]. Given the technical and practical difficulties associated with the creation of transgenic mosquito strains [61], as well as the fact that some of the more common *Drosophila* hemocyte markers are not encoded in the mosquito genome (e.g., hemese), the CM-DiI approach described here for the first time allows the study of mosquito hemocyte cell biology in vivo and in real time. We expect that this procedure could be adapted for the study of hemocyte biology in a broad range of insects.

In conclusion, there remain deficits in our current knowledge of hemocyte biology in adult insects, as well as in our understanding of the direct interactions between the insect circulatory and immune systems. Here, we developed new methods for the in vivo study of mosquito hemocytes and pericardial cells (nephrocytes), and applied these methods to discover a novel mosquito immune response. Namely, we uncovered periostial hemocyte aggregates, an immune tissue that is located on the surface of the mosquito heart and represents a basal component of the cellular immune response against bacteria and malaria parasites.

## Materials and Methods

### Ethics statement

This study was carried out in strict accordance with the recommendations in the Guide for the Care and Use of Laboratory Animals of the National Institutes of Health, U.S.A. The protocol was approved by Vanderbilt University's Institutional Animal Care and Use Committee (IACUC; VU animal use protocols M/10/381 and M/08/041). Animals were maintained in a certified animal room and were cared for by trained personnel and veterinarians.

### Mosquito rearing and maintenance

*Anopheles gambiae* (G3 strain) were reared and maintained in an environmental chamber as described [3]. Briefly, larvae were hatched in plastic containers and fed a mixture of koi food and yeast. Pupae were separated by size, allowed to develop into adults, and maintained on a 10% sucrose solution at 27°C, 75% relative humidity and a 12 h light/12 h dark photoperiod. Unless stated otherwise, all experiments were carried out on female mosquitoes at 5 days post-eclosion.

### Mosquito injections, bacterial infections, and treatment with immune elicitors

For injections, mosquitoes were cold anesthetized and a finely pulled glass needle was inserted through the thoracic anepisternal cleft. A volume of 0.2 µl was slowly injected into the hemocoel and mosquitoes were then placed back in an environmental chamber until assayed.

For bacterial infections, tetracycline-resistant GFP-expressing *E. coli* (modified DH5α) were grown overnight in a shaking incubator at 37°C in Luria-Bertani's rich nutrient medium (LB broth) and, unless otherwise stated, cultures were normalized to OD_600_ = 2 or OD_600_ = 4 using a BioPhotometer plus spectrophotometer (Eppendorf AG, Hamburg, Germany) prior to injection. To determine the absolute infection dose, dilutions of each OD_600_ = 2 and OD_600_ = 4 *E. coli* culture were plated on LB agar with tetracycline, incubated at 37°C, and the resultant colony forming units were counted 18 h later. On average, OD_600_ = 2 and OD_600_ = 4 represented infection doses of 38,000 and 131,000 *E. coli* per mosquito, respectively. As a non-living phagocytosis elicitor [11], 1 µm diameter FluoSpheres carboxylate modified microspheres (Molecular Probes; Eugene, OR) were also injected into mosquitoes. Microspheres were mixed with phosphate buffered saline (PBS; pH 7.0) to a final concentration of 0.08% solids per volume prior to injection.

Three soluble immune elicitors were used in this study: peptidoglycan (PGN) purified from Gram(−) *E. coli*, PGN purified from Gram(+) *Bacillus thuringiensis*, and β-1,3-glucan. For PGN purification, *E. coli* and *B. thuringiensis* were grown overnight in LB broth at 37°C. A volume of 2 ml of each bacterial culture was independently centrifuged for 1 min at 10,000 rcf, and the resulting pellets were suspended in 1 ml of PBS. Bacteria were then lysed, while on ice, by sonication for 1 min using a Branson Sonifier 450 (Branson Ultrasonics; Danbury, CT) equipped with a 3 mm tip that was set to 20% power and 30% duty cycle. Trichloroacetic acid (TCA) was added to the lysed bacteria to a final concentration of 10% v/v, the resultant solutions were incubated for 10 min at 90°C, and the PGNs were extracted by centrifugation for 1 min at 12,000 rcf [62]. The PGN pellets were then washed 3 times by resuspending in 1 ml 75% ethanol and centrifuging for 1 min at 12,000 rcf. After the final centrifugation the PGNs were resuspended in 1 ml PBS, and the purified PGNs were dissociated into soluble fragments while on ice by sonicating for 30 min at 20% power and 30% duty, with 50% rest periods every 40 sec. Following dissociation, all non-soluble material was removed by centrifugation per standard protocol [62], and PGN solutions were normalized to OD_600_ = 1 prior to injection.

β-1,3-glucan (microparticulate curdlan), a fungal immune elicitor, was prepared by sonicating 1% w/v curdlan (Sigma-Aldrich; St. Louis MO) in PBS for 5 min at 20% power and 20% duty, while on ice. Particulates remaining in the solution were then allowed to settle for 30 min while on ice, and the clear top phase was removed and used for injections [63]. Finally, although lipopolysaccharide (LPS) is commonly used as an immune elicitor, it was excluded from this study because it has been shown that LPS has little or no immunostimulatory effect in non-mammalian animals [64], [65]. Regardless, preliminary experiments using LPS yielded results that were qualitatively similar to the PGN experiments, but these observations could be due to the residual PGN found in LPS preparations.

### Blood feedings and *Plasmodium berghei* infections

Five-day-old female adult mosquitoes were starved for 6 h and then allowed to feed on a *P. berghei*-infected mouse with approximately 10% blood-stage parasitemia and a 1–2% gametocytemia. A control group of mosquitoes originating from the same cohort was allowed to feed, concurrently, on an uninfected mouse. Both groups were then housed in a humidified chamber at 20.5°C for 20 days prior to the assessment of the periostial cellular immune response (see below). The PbGFP_CON_ *P. berghei* strain [66] was used for experiments where periostial hemocyte numbers were counted. The RedStar strain [67] was used to observe the interaction between hemocytes and sporozoites, as this strain retains a higher level of fluorescence following aldehyde fixation. The infection status of each mosquito was determined by visualizing parasites in the midgut and the salivary glands.

### In vivo staining of mosquito hemocytes and hemolymph perfusions

To stain hemocytes inside live mosquitoes, 0.2 µl of a solution consisting of 75 µM CM-DiI (Vybrant CM-DiI Cell-Labeling Solution, Invitrogen) and 0.75 mM Hoechst 33342 (Invitrogen) in PBS was injected into mosquitoes. It was crucial that this solution be injected within minutes of its preparation, as once the CM-DiI is placed in an aqueous environment its hemocyte-staining effectiveness rapidly decreases, approaching 0% after 10–15 min of mixing. After CM-DiI injection, mosquitoes were immediately returned to 27°C and 75% relative humidity for an incubation period of 20 min.

Circulating hemocytes were collected by perfusing the hemolymph onto the center of 1 cm diameter etched rings on Rite-On (Gold Seal; Portsmouth, N.H.) glass slides [25]. Cells were allowed to adhere to slides for 20 min at room temperature, fixed for 20 min with 4% formaldehyde in PBS, washed 3 times for 5 min with PBS, and coverslips were mounted with Aqua Poly/Mount (Polysciences; Warrington, PA). Visual examination of adherent perfused hemocytes was conducted using a Nikon 90i compound microscope (Nikon; Tokyo, Japan) equipped with a Nikon Intensilight C-HGFI fluorescence illumination unit and a CoolSNAP HQ^2^ digital camera (Roper Scientific; Ottobrunn, Germany). Cells were counted at 1000× magnification by scanning the slides from the far left to the far right until 50 hemocytes from each individual mosquito were visualized, also keeping track of the number of fat body cells observed. Intact cells were first identified as either hemocytes or fat body by confirming the presence of a nucleus using fluorescence microscopy (Hoechst 33342) and comparing cell morphology by differential interference contrast (DIC) microscopy to the descriptions of previous authors [18]. Specifically, hemocytes are significantly smaller than fat body cells and do not contain large lipid droplets. Hemocytes were then examined for the presence of phagocytosed GFP-expressing *E. coli*, and all cell types were examined for their incorporation of CM-DiI. Three treatments were performed (naïve, LB injected and *E. coli* injected), and for each treatment, hemocytes from 15 individual mosquitoes that originated from 5 independent but paired cohorts were examined (i.e., for each treatment, 3 mosquitoes per cohort).

### Staining of pericardial cells, heart muscle, and sessile hemocytes

The dorsal portion of mosquito abdomens were analyzed after the labeling of PCs, hemocytes and heart muscle. These tissues were labeled in the presence or absence of an immune challenge, and were labeled in the following combinations: (1) PCs and heart muscle, (2) PCs and hemocytes, and (3) hemocytes and heart muscle. In all experiments, Hoechst 33342 was used as a nuclear stain.

Depending on the experiment, PCs were stained using one of two novel methods. In the first, more permanent method, 0.2 µl of 0.2 mg/ml Alexa Fluor conjugated IgG (either 488 or 568 nm; Molecular Probes) in PBS was intrathoracically injected and the mosquitoes were placed at 27°C for 1 h to allow the labeled proteins to be pinocytosed by the PCs. The mosquitoes were then placed in PBS and their abdomens were bisected along a coronal plane. The dorsal half of each abdomen, sans any internal organs, was isolated, rinsed, fixed in 4% formaldehyde in PBS for 10 min, washed 3×5 min in 0.1% Tween 20 in PBS (PBST), and mounted on a glass slide using Aqua Poly/Mount. For the second PC staining method, 0.2 µl of a 0.1 mM mixture of LysoTracker Red (Molecular Probes) in PBS was intrathoracically injected and allowed to incubate for 10 min. Abdomens were then bisected along a coronal plane, rinsed in PBS, and mounted using Aqua Poly/Mount. Because LysoTracker Red stains any region that contains high lysosomal activity, it also serves as a marker for hemocyte-mediated phagocytosis of bacteria. However, LysoTracker Red cannot be aldehyde-fixed, so this method stains the PCs briefly before the dye diffuses out, and is not useful in combination with most other staining techniques.

To examine and quantify hemocytes that were adhered to tissues, 0.2 µl of a solution consisting of 75 µM CM-DiI and 0.75 mM Hoechst 33342 in PBS was injected into mosquitoes. After allowing this solution to incubate in live mosquitoes for 20 min at 27°C, mosquitoes were injected with 0.2 µl of 16% formaldehyde. Tissues were then allowed to fix for 5 min, the mosquitoes were bisected along a coronal plane, and the dorsal halves were mounted on glass slides using Aqua Poly/Mount. Immediately following mounting, CM-DiI stained hemocytes in the periostial regions were counted through the 90i's oculars at 400× or 1000× total magnification. Hemocytes were only counted if their presence was supported by both CM-DiI and Hoechst 33342 staining, so a small number of cells (<5%) might have been excluded from our counts using this conservative method. Cells were counted as periostial hemocytes only if they were attached to the dorsal vessel at the ostia, or formed part of a contiguous mass of hemocytes that were attached to this region. Numbers of periostial hemocytes were recorded on a per mosquito basis, and for each treatment cell counts were conducted on at least 12 mosquitoes that originated from no fewer than 4 independently-reared cohorts. In these experiments, trials were excluded when background staining interfered with the cellular boundaries of hemocyte aggregates in any of the treatment groups.

For the co-staining of PCs and heart muscle, 1 h after the injection of Alexa Fluor-conjugated IgG (568 nm) muscle was stained by injecting a formaldehyde-phalloidin-Hoechst-Triton×100 mixture as described [3]. Abdomens were then washed by perfusion with PBST 3 times for 5 min each, fixed a second time using 4% formaldehyde, and bisected and mounted as above. For the co-staining of PCs and hemocytes, Alexa Fluor-conjugated IgG (488 nm) was injected to stain the PCs, and after 1 h the hemocytes were stained with CM-DiI as described above. For the co-staining of hemocytes and heart muscles, CM-DiI was injected and allowed to incubate for 20 min at 27°C, and 0.2 µl of 16% formaldehyde was then intrathoracically injected to kill and preserve the mosquito. Abdomens were then bisected, suspended for 15 min in a Phalloidin-Hoechst-Triton×100 mixture [3], washed, and mounted on glass slides using Aqua Poly/Mount.

### Still image acquisition

Perfused hemocytes and abdominal whole mounts were imaged between 200× and 1000× magnification depending on which phenomenon was being captured. Specimens were viewed under differential interference contrast microscopy (DIC) and/or fluorescence illumination using the Nikon 90i microscope ensemble described above, and Z-stack images were captured using a linear encoded Z-motor and Nikon's Advanced Research NIS-Elements software. To display 2 dimensional images, all images within a stack were combined to form a focused image using the Extended Depth of Focus (EDF) module of NIS Elements. For 3 dimensional rendering, Z-stacks were quantitatively deconvolved using the AQ 3D Blind Deconvolution module of NIS Elements and rendered using the volume view feature.

To quantify relative levels of *E. coli* fluorescence in the abdomen following infection, a series of images were captured and analyzed using the fixed thresholding feature in NIS-Elements. All images were acquired under non-saturating conditions using identical settings, and were identically thresholded to isolate only the portions containing GFP-*E. coli* fluorescence. For each specimen, fluorescence intensity data was collected for the dorsal abdomen, the entire abdomen (dorsal+ventral), and the periostial regions.

### Intravital time-lapse video microscopy

For intravital time-lapse video recording of the interactions between hemocytes, pathogens and the mosquito heart, hemocytes were labeled in vivo using the CM-DiI method described above. Mosquitoes were then restrained on glass slides using small strips of Parafilm “M” (Pechiney Plastic Packaging, Chicago) to gently adhere the proboscis, legs and wings (extended) to glass slides, and spheres of Parafilm were placed on either side of the abdomen to restrict side-to-side movement. Once mosquitoes were restrained, GFP-expressing *E. coli* were injected and fluorescence-based intravital video recording of the heart region was initiated within 10 sec of treatment. Time-lapse image sequences were captured for green (GFP-*E. coli*) and red (CM-DiI-stained hemocytes) channels at 5 sec intervals using the ND-experiment capture module of NIS Elements. Hemocyte tracking was then done using the manual feature of the Object Tracker module of NIS-Elements, and hemocyte velocity was calculated by dividing the path length by the total time of tracking. To reduce the potential for damage to the mosquito, light shutters were only open while each image was being acquired, and light intensity was greatly reduced by using a neutral density filter of 16.

### Statistical analyses

Cell count data was separated by treatment group and tested for normality using the Kolmogorov-Smirnov test. After confirming normality, datasets with one variable and two groups were analyzed by the t-test. Datasets with one variable and more than two groups were analyzed by one-way ANOVA, and multiple comparisons were done using Tukey's post hoc test. Differences were deemed significant at P<0.05.

For statistical evaluation of the co-localization of *E. coli* fluorescence (GFP) and hemocyte fluorescence (CM-DiI) within perfused hemocyte samples or whole mount abdomens, images were analyzed using the Mander's overlap or Pearson's correlation coefficient (PCC) features in NIS-Elements. These measures are similar in that they describe the amount of spatial overlap between two fluorescence channels, with the main difference being that Mander's overlap corrects for differences in signal intensity while PCC does not [68].

## Supporting Information

Movie S1 Phagocytosis of *E. coli* by periostial hemocytes.
(MOV)

Movie S2 Hemocyte migration to the periostial regions in response to *E. coli* infection.
(MOV)
